# Longitudinal Brain Atrophy Rates in Transient Ischemic Attack and Minor Ischemic Stroke Patients and Cognitive Profiles

**DOI:** 10.3389/fneur.2019.00018

**Published:** 2019-02-19

**Authors:** Muhammad Munir, Jake Ursenbach, Meaghan Reid, Rani Gupta Sah, Meng Wang, Amith Sitaram, Arooj Aftab, Sana Tariq, Giovanna Zamboni, Ludovica Griffanti, Eric E. Smith, Richard Frayne, Tolulope T. Sajobi, Shelagh B. Coutts, Christopher D. d'Esterre, Philip A. Barber, Alzheimer's Disease Neuroimaging Initiative

**Affiliations:** ^1^Calgary Stroke Program, Department of Clinical Neurosciences, Foothills Medical Centre, Calgary, AB, Canada; ^2^Seaman Family MR Center, Foothills Medical Centre, Calgary, AB, Canada; ^3^Department of Radiology, Foothills Medical Centre, Calgary, AB, Canada; ^4^Cumming School of Medicine, Hotchkiss Brain Institute, University of Calgary, Calgary, AB, Canada; ^5^Department of Community Health Sciences, University of Calgary, Calgary, AB, Canada; ^6^Nuffield Department of Clinical Neurosciences, FMRIB Centre, University of Oxford, John Radcliffe Hospital, Oxford, United Kingdom

**Keywords:** brain, transient ischemic attack, stroke, cognition battery, atrophy rates, diffusion weighted imaging, white matter lesion, longitudinal

## Abstract

**Introduction:** Patients with transient ischemic attack (TIA) and minor stroke demonstrate cognitive impairment, and a four-fold risk of late-life dementia.

**Aim:** To study the extent to which the rates of brain volume loss in TIA patients differ from healthy controls and how they are correlated with cognitive impairment.

**Methods:** TIA or minor stroke patients were tested with a neuropsychological battery and underwent T1 weighted volumetric magnetic resonance imaging scans at fixed intervals over a 3 years period. Linear mixed effects regression models were used to compare brain atrophy rates between groups, and to determine the relationship between atrophy rates and cognitive function in TIA and minor stroke patients.

**Results:** Whole brain atrophy rates were calculated for the TIA and minor stroke patients; *n* = 38 between 24 h and 18 months, and *n* = 68 participants between 18 and 36 months, and were compared to healthy controls. TIA and minor stroke patients demonstrated a significantly higher whole brain atrophy rate than healthy controls over a 3 years interval (*p* = 0.043). Diabetes (*p* = 0.012) independently predicted higher atrophy rate across groups. There was a relationship between higher rates of brain atrophy and processing speed (composite *P* = 0.047 and digit symbol coding *P* = 0.02), but there was no relationship with brain atrophy rates and memory or executive composite scores or individual cognitive tests for language (Boston naming, memory recall, verbal fluency or Trails A or B score).

**Conclusion:** TIA and minor stroke patients experience a significantly higher rate of whole brain atrophy. In this cohort of TIA and minor stroke patients changes in brain volume over time precede cognitive decline.

## Introduction

Transient ischemic attack (TIA) and minor stroke, traditionally considered as risk factors for major ischemic stroke, are common medical emergency (incidence 0.37–1.1 per 1,000) associated with a four-fold increase risk of cognitive decline and dementia (1–3). Mechanisms linking TIA and minor stroke with cognitive decline are poorly understood. More than a third of patients with TIA have impairment of more than one cognitive domain within 3 months of their TIA that is not entirely explained by silent brain infarcts (4). Pathological studies link late life cognitive impairment and brain atrophy to two dominant disease entities, Alzheimer's disease (AD) and small vessel disease (5, 6). Fifty per cent of late-life cognitive impairment is attributed to vascular and lifestyle-related risk factors in midlife. Still unknown are the incipient disease processes which may contribute to cognitive dysfunction, and whether these can be modified through vascular risk reduction strategies guided by biologically relevant markers of disease progression (7). For instance, early macroscopic brain loss (i.e., volume loss in ml/year), measured by serial magnetic resonance imaging (MRI), has been shown to be a predictor of cognitive decline in AD and mild cognitive impairment (8). Yet, studies identifying early incipient disease stroke and TIA cohorts vary considerably in clinical selection criteria, cognitive measures and methods for measuring changes in brain structure over time (9–11).

In this longitudinal cohort study of non-demented TIA/minor stroke patients, we hypothesize the TIA/minor stroke cohort will have increased rates of cerebral brain atrophy (annualized percent brain volume change, PBVC) compared to healthy controls (HC) measured with repeated high-resolution MR imaging. We aim to correlate PBVC with change in neuropsychological performance over a 3 years period.

## Materials and Methods

### Study Population

Patients were recruited from the Extended-CATCH (*C*T *a*nd MRI in the *T*riage of TIA and minor *C*erebrovascular events to identify *H*igh risk patients) study (12) at the Foothills Medical Center or Calgary Stroke Prevention Clinic from March 2009 to December 2012, while healthy controls were drawn from the Alzheimer's Disease Neuroimaging Initiative (ADNI) study. TIA was diagnosed by disruptions to speech and motor symptoms that lasted longer than 5 min but <24 h. Minor ischemic stroke was defined by a National Institute of Health Stroke Scale (NIHSS) score of <4. Demographic and medical information was collected including vascular risk factors such as blood pressure, body mass index (BMI), history of hypertension and diabetes. Hypertension positive was defined as past medical history of hypertension or is currently prescribed anti-hypertension medications. MRI was acquired 48 h of symptoms (baseline), at 18 months and 3 years. The MRI protocol at each time point included a high-resolution T1-weighted and 3D FLAIR sequences as described below.

Inclusion criteria for Ex CATCH TIA/minor stroke cohort included: (1) age 50–80 years; (2) established vascular risk factors; and (3) the acquisition of serial brain MRI scans at all 3 time points. Exclusion criteria for Ex CATCH TIA/minor stroke cohort included: **(**1) dementia as defined by the National Institute of Aging-Alzheimer Association Criteria (13); (2) other central nervous system diseases (e.g., MS), alcoholism, substance abuse, sedatives, antipsychotic medications and history of psychiatric illness that might impact cognitive testing and follow-up; **(**3) Other comorbidities that could significantly interfere with cognitive performance and functional outcome (e.g., recent coronary bypass surgery, comorbidities), English as a second language, and the inability to complete neuropsychological testing. The University of Calgary Conjoint Health Research Ethics Board formally approved the study, and all patients provided written informed consent prior to participation.

The healthy control data used in the preparation of this article were obtained from the Alzheimer's Disease Neuroimaging Initiative (ADNI) database (adni.loni.usc.edu). The ADNI was launched in 2003 as a public-private partnership, led by Principal Investigator Michael W. Weiner, MD. The primary goal of ADNI has been to test whether serial MRI, positron emission tomography (PET), other biological markers, and clinical and neuropsychological assessment can be combined to measure the progression of mild cognitive impairment (MCI) and early Alzheimer's disease (AD). For up-to-date information, see www.adni-info.org. Well-characterized healthy control data was obtained through ADNI, made permissible through approval by the ADNI collaborators. The use of ADNI data for scientific investigations has been established by other studies that compare specific disease groups with the ADNI subjects (14–16).

ADNI inclusion criteria for healthy controls included a Mini Mental State Examination (MMSE) score between 24 and 30, a Clinical Dementia Rating of 0, no indication of depression, MCI, or dementia, cognitively normal, modified Hachinski score ≤ 4, Geriatric Depression Scale < 6, and normal memory function. ADNI exclusion criteria included significant neurological disease, MR evidence of infection, infarction, or lesions, inability to complete MRI due to medical devices in the body, psychiatric disorders, history of alcohol or substance abuse, any significant illness or medical instability, use of psychoactive medication.

### Clinical Data Collection

At study entry baseline evaluation included clinical review, fasting cholesterol, glucose, and renal function. Treatments for hypertension, diabetes, hyperlipidemia, anti-thrombotic agents, and other medications that could influence cognition such as sedatives, anxiolytics, or psychotropic medications are collected. We screened for obstructive sleep apnea (17). Blood pressure (BP) measurement was recorded from average of 3 sitting BPs at baseline and each subsequent visit. All participants are managed according to current stroke prevention guidelines (18).

### Image Acquisition

#### Extended CATCH TIA/Minor Stroke Cohort

To evaluate whole brain atrophy rates, TIA and minor stroke patients underwent a T1-weighted volumetric acquisition using a 3T scanner (Signa NV/I or Discovery 750; General Electric Healthcare, Waukesha, WI). High-resolution T1 images were acquired at baseline (within 48 h of symptoms) and at follow-up (TE/TR = 2.73 ms/7.0 ms, flip angle = 8°, TI = 650 ms, acquisition matrix = 256 × 256). 3D FLAIR (TE/TR/flip angle 140 ms/ 9,000 ms/90°, acquisition matrix = 256 × 256) were acquired at the same time points. Whole brain atrophy rates were measured between baseline (within 48 h of stroke symptoms) and 18 months (Interval 1), and 18 months to 3 years (Interval 2). In the middle of our study, a hardware upgrade occurred to a GE Discovery 750 scanner preventing whole brain atrophy rates being calculated at Interval 1 in those cases where MRI data was acquired before the system upgrade. Quality assurance to detect image distortion was conducted to assess the adequacy of key image properties including image uniformity, image contrast, and signal-to-noise. This visual imaging inspection was performed on every scan and exclusion criteria and image quality was graded according to neck and head movement, quality of registration and intensity inhomogeneities.

For Extended CATCH subject a stroke neurologist and neuroradiologist reviewed all images. Acute infarct location was recorded as follows: (1) cortical (2) deep (basal ganglia, internal capsule, thalamus, and deep white matter tracts) (3) cortical and deep, and (4) infratentorial. DWI lesion volume were calculated using Quantomo (Cybertrials Inc., Calgary, Canada), and have been previously reported (19). For the segmentation of white matter hyperintensities (WMHs) baseline and follow up fluid-attenuated inversion recovery (FLAIR) images were registered to the high-resolution T1 images using a rigid transformation and cost function of the mutual information. WMH volume (corrected for total intracranial volume) were calculated using Cerebra-WML software, a semi-automated software measured the volume (ml) of white matter lesions segmentation is based on the global threshold and contrast between the different anatomical regions of the brain (20), following international recommendations for the reporting, image acquisition and analysis of small vessel disease reported by our group (21, 22). To evaluate the accuracy of these WM segmentations, two trained clinicians conducted quality control of the semi- automated segmentations by visually inspecting all the FLAIR images.

#### ADNI Healthy Controls

ADNI controls were imaged using the standardized ADNI protocol (adni.loni.usc.edu/methods/documents/mri-protocols) (23). Initial inspection of the ADN1-and ADNI GO database revealed 417 healthy controls, and then identified 70 healthy controls that met the age inclusion criteria, and had undergone 3T MRI at baseline, and follow up at years 1 and 3 years ± 6 months. 3T MRI data was download from the ADNI website and included MRIs from Siemens Medical Solutions, Phillips, and General Electric Healthcare. MR protocols included the acquisition of sagittal high-resolution volumetric T1-weighted, inversion recovery prepared, structural images, and 3D FLAIR. MPRAGE (or vendor equivalent) high resolution T1 sequences were acquired (TE = min full echo, TR + 2,300, TI = 900 ms, acquisition matrix = 256 × 256 @1 × 1 × 1 mm) and SD FLAIR (Effective TE = 119, TR = 4,800, T1 = 1,650, acquisition matrix = 256 × 256 @ 1.2 × 1 × 1 mm).

### Image Analysis

#### Measurement of Baseline Brain, Cortical Gray Matter, and White Matter Volume

SIENAX (Structural Imaging Evaluation Using Normalization of Atrophy, Cross-sectional) was used to measure the brain volume, normalized for head size, cortical gray and white matter volume (24, 25). Image analysis using SIENAX consists of 4 main steps: brain extraction, registration to MNI152 standard template, standard-space masking, tissue-type segmentation, and calculation of total brain volume.

#### Measurement of Whole Brain Atrophy Rates

A detailed outline of the procedure is provided elsewhere (24, 25). Measurement of whole brain atrophy rates was conducted using SIENA (24). Each MRI underwent an imaging quality control, which included ensuring proper brain extraction results that did not include non-brain tissue, proper registration to MNI152 template and accurate segmentation of the whole-brain. Briefly, the surface of the brain and the internal brain/cerebrospinal fluid boundaries were identified automatically (Structural Image Evaluation, using Normalization, of Atrophy, SIENA, part of FMRIB Software Library, FSL; https://www.fmrib.ox.ac.uk/fsl), followed by inspection and manual correction (26). These corrections included distortion due to gradient non-linearity; for image intensity non-uniformity (N3); for B1 non-uniformity where required; and scaling based on ADNI phantom measures (23). The 18 months and 3 years high resolution T1-weighted acquisition were also registered to the baseline T1-w acquisition via neighborhood-normalized cross-correlation.

### Neuropsychological Assessment

TIA and minor stroke patients underwent neuropsychological testing 90 days post ictus, and then annually for 3 years. A modified version of the Canadian Stroke Network and National Institute on Neurological Disorders and Stroke Vascular Cognitive Impairment battery was used (27). The battery tested frontal/executive, memory, language, and visuospatial domains, and required ~60–70 min to administer. Specifically, the tests used were: Trail making Test (Parts A and B) (28), WAIS-III Digit Symbol Coding (29), Controlled Oral Word Association Task (30), Boston Naming Test second edition—short form (31), California Verbal Learning Test—II (32), Rey Osterrieth Complex Figure Task (33), Boston Diagnostic Aphasia Examination third edition—Complex Ideational Material subtest (34), and CLOX1 clock drawing task (35). Domain-specific composite z-score variables were created: (1) EF (average of Trails B and COWAT FAS), (2) psychomotor processing speed, PS (average of Trails B and Digit Symbol Coding), and (3) verbal memory-Memory: CVLT List A Delayed Free Recall which was calculated by taking the average of [2/3(average of trials 1–5) +1/3(trials 12 and 12 false positives). Performance on composite measures for each patient (19). Median (IQR) is reported in the Table S1.

### Statistical Analysis

Differences between TIA and minor stroke patients and ADNI healthy controls demographic and clinical characteristics were tested using *t*-test or Fisher's exact tests. An analysis of variance was performed using all time points within each neuropsychological test. A linear mixed effects regression model with a random intercept was used to compare longitudinal changes in brain atrophy between patients and healthy controls, adjusting for demographic characteristics and vascular risk factors. The linear mixed-effects regression model considers the effects of both the within-subject and between-subject variation. For example, a TIA/minor stroke patient can respond differently over a longitudinal study with respect to brain volume (within-subject) and the variation in response of each individual patient may be different from another patient (between-subject). In our study, we anticipated a linear relationship between the independent and dependent variables. This relationship is characterized by the linear coefficient, which highlights the trend of the response variable with the predictor. Linear regression helps model the outcome and response variables while still incorporating patients who may vary in their independent variable. The model is best suited to longitudinal studies as it can incorporate missing values at specified time points of the study design, such as missing imaging or neuropsychological.

Our analysis consisted of two parts: (1) to determine whether Extended CATCH patients had increased whole brain atrophy rates, a linear mixed-effects regression analysis was employed to model atrophy rates between Extended-CATCH with ADNI. The model incorporated independent covariates that may independently modify whole brain atrophy rates such as age (years), sex (female), time (years), and vascular risk factors including history of hypertension, smoking, white-matter hyperintensity volume (mL) and diabetes; (2) a series of mixed effects regression models were estimated to determine the relationship between baseline MRI (whole brain volume, gray matter volume, white matter volume, and white matter hyperintensity volume), and annualized percentage volume change (PBVC) of whole brain with cognitive functioning defined by the selected cognitive tests and for three cognitive domains (executive function, processing speed and memory).

In the first part of the analysis, imaging time interval was a dichotomous variable coded zero if the atrophy rate measurement spanned 24 h to 18 months for patients or baseline to 1 year for healthy controls (labeled as the first interval). It was coded one if the atrophy rate measurement spanned 18 months to 3 years for TIA/minor stroke patients or 1 year to 3 years for ADNI data (labeled as the second interval). All atrophy rates were annualized to account for differences in interval duration.

In the second part of the analysis, there was no comparative neuropsychological data for the ADNI controls, in the TIA/ minor stroke cohort only, a series of linear mixed effects models were used to estimate longitudinal cognitive test performance vs. annualized rate of brain atrophy, while controlling for demographic and medical characteristics, and including a random slope and intercept for each participant. Covariates in the cognitive outcome models are: Time of cognitive testing (years, ~4 measurements beginning 90 days post-ictus and annually for 3 years thereafter), age at first assessment (years), Education (years), initial Diffusion Weighted Imaging Lesion Volume (mL), baseline White Matter Hyperintensity Volume (mL), baseline Gray Matter Volume, and baseline White matter volume. As atrophy rate intervals (48 h, 18 months, 3 years) did not align with cognitive testing (90 days, 1 year, 2 years, 3 years), they were estimated as a time-varying covariate and grouped as follows: interval 1 PBVC (48 h to 18 months) with first two cognitive testing time points (90 days, 1 year); interval 2 PBVC (18 months to 3 years) with second two cognitive testing time points (2 years, 3 years). To determine if the results were sensitive to grouping of these variables, the 1 year and 2 years cognitive testing time points were grouped with the opposite intervals in two models, however no difference in results was noted.

In all mixed effects models described above, a subject-specific intercept and slope was estimated for each participant. Mixed effects regression permits modeling of longitudinal outcomes using all available data, for example, if a participant is missing the cognitive assessment at 2 years, a slope and intercept for them would still be estimated based on their cognitive performance at 90 days, 1 year, and 3 years while controlling for atrophy rate and covariates. In a process analogous to meta-analysis, these subject-specific regression lines are summarized across participants while accounting for the added error due to missing data points (36). Statistical analysis was carried out using R (version 3.3.2, R Core Team) using the package LME4 (36) and *p* < 0.05 was used to determine statistical significance, except on the analysis of cognitive performance, where a Bonferroni correction was applied to control for inflation of the family-wise error rate producing a more conservative significance threshold of *p* < 0.006. All mixed effects models were fitted using Restricted Maximum Likelihood estimation. Where appropriate means ± standard deviations are reported.

## Results

### Patient Demographics, Imaging and Neuropsychological Outcomes

A total of 90 patients were recruited to the Extended-CATCH study and those patients with 2 consecutive MRI scans by March 2014 were included into the study. Over the course of 3 years, 2 patients were deceased, 8 withdrew from the study, resulting in 80 patients (10% attrition) completing the study at 3 years. A total of 78 had baseline MRI, and cognitive assessment at least two time points. Demographic data and vascular risk factors for TIA and minor stroke patients, and healthy control participants are summarized in Table 1. On average, the TIA and minor stroke patient group were younger than the ADNI healthy control group (*p* < 0.001), contained a higher proportion of men (*p* < 0.001), those with hypertension (*p* = 0.005), and current smokers (*p* = 0.002). The TIA and minor stroke patients had significantly higher white matter hyperintensity volume (*p* < 0.001). Neuropsychological testing was performed for the patient group on average 111 ± 24 days' post-event, and then annually for 3 years (year 1 = 430 ± 76 days; year 2 = 746 ± 43 days; year 3 = 1,130 ± 43 days). In the Ex CATCH cohort there was a significant improvement in composite memory and CVLT scores, and digit symbol coding between baseline and 3 years (Table S1).

**Table 1:** Demographic and medical characteristics of TIA and minor stroke patients and healthy controls.

**Characteristics**	**Patient group**	**Healthy control group**	***p***
Number of subjects, *n*	80	70	
Age, years	64.5 ± 12.7	70 ± 4.4	<0.001
Gender, males (%)	63 (78.8%)	32 (45.7%)	<0.001
Hypertension, *n* (%)	42 (52.5%)	20 (28.6%)	0.005
Diabetes, *n* (%)	11 (13.8%)	3 (4.3%)	0.053
BMI, kg/m^2^ (*SD*)	27.2 ± 4.1	26.8 ± 6.8	0.685
Current Smoker, *n* (%)	10 (12.5%)	24 (34.3%)	0.002
White matter hyperintensity volume, mL (*SD*)	10.2 ± 8.8	5.3 ± 6	<0.001
Annualized rate of atrophy at first interval (*SD*)	−0.636 ± 0.552	−0.547 ± 0.813	0.503
Annualized rate of atrophy at second interval (*SD*)	−0.966 ± 0.812	−0.583 ± 0.498	0.001
Normalized Brain volume, mL (*SD*)	1400.4 ± 87.2	1,421 ± 79.1	0.551
Gray matter brain volume, mL (*SD*)	724 ± 54.8	730.3 ± 46.3	0.847
White matter volume, mL (SD)	675.5 ± 44.8	691.5 ± 45.5	0.316

### Atrophy Rates

Whole Brain atrophy rates were calculated for 80 unique participants: 39 participants over the first interval, and 72 participants over the second interval. One atrophy measurements over the first interval and 4 over the second-time interval were excluded because one or both T1-weighted volumetric images did not meet quality control criteria. Therefore, 38 subjects were included in Interval 1 and 68 subjects in Interval 2. Twenty-eight subjects had available MRI data from Interval 1 and 2. For the ADNI group, 70 healthy controls were included in the study. A total of 210 T1-weighted volumetric images were included: 70 at baseline; 70 at 1 year; and 70 were available at 3 years.

Among patients over the first interval, a group mean whole brain atrophy rate of −0.64 ± 0.55% was documented, compared with −0.54 ± 0.32% for the healthy controls *(p* = 0.33). Over the second interval, patients demonstrated a whole brain atrophy rate of −0.97 ± 0.81% compared with healthy controls at −0.58 ± 0.50% (*p* < 0.001). Overall, when pooled over both intervals, in TIA/minor stroke patients the annualized whole-brain atrophy rate was −0.85% ± 0.74 while in ADNI the annualized whole-brain atrophy was −0.53% ± 0.38 *(p* < 0.001).

Table 2 shows the association between annualized rate of brain atrophy and group effect (TIA vs. controls) controlling for subject demographic and vascular characteristics. TIA/minor stroke patients demonstrated a higher annualized rate of brain atrophy than ADNI controls. On average, patients with diabetes demonstrated significantly higher levels of brain atrophy than those without. But there was no evidence of any statistical significant effect of imaging time interval, sex, hypertension, BMI, smoking status or the baseline, WMH volume on annualized rate of brain atrophy. As there appeared to be no relationship with WMH volume and rates of whole brain atrophy, additional statistical analysis of sub-classification of WMH with brain atrophy or cognition was not performed.

**Table 2:** Annualized whole brain atrophy rate of TIA and minor ischemic stroke participants and healthy controls.

	**Unstandardized regression coefficient**	**95% CI**	***p***
Group (CATCH = 1, ADNI = 0)	−0.228	[−0.462, −0.016]	0.043
Time (first interval = 0; second interval = 1)	−0.155	[−0.324, 0.016]	0.09
Age (years)	−0.01	[−0.021, 0]	0.051
Gender (female)	−0.018	[−0.224, 0.147]	0.854
Diabetes	−0.419	[−0.716, −0.085]	0.012
Hypertension	0.103	[−0.115, 0.292]	0.295
Current smoker	0.035	[−0.2, 0.253]	0.745
WMH Volume (mL)	−0.013	[−0.025, −0.001]	0.057

*WMH, white matter hyperintensity. The unstandardized regression coefficients (estimate) are provided for each covariate*.

The relationship between neuropsychological test performance and atrophy rate is shown in Table 3. Each row in Table 3 denotes a separate mixed-effects regression model where the indicated cognitive outcome is estimated longitudinally by atrophy rate and covariates. Only the regression weights, confidence intervals, and *p*-values for atrophy rate are reported, while the covariates are not. Table 3 shows that there is a modest relationship with higher rates of brain atrophy and processing speed composite and digit symbol coding but no relationship was seen between brain atrophy rates and memory, executive or other individual cognitive test scores (CVLT, Boston Naming, Trails A and B, verbal fluency). After correcting for multiple comparisons, PBVC was not significantly associated with cognitive functioning above and beyond the effects of time, age, DWI lesion volume, education, white matter hyperintensity volume, baseline gray matter volume, and baseline white matter volume. Neither was there a relationship with baseline WMH volume, and normalized brain volume (or gray and white matter volume) compared to cognitive outcomes after applying Bonferroni correction to the *p*-value. With regard to covariate significance after applying the Bonferroni correction, the participants' scores on the Memory Composite Index [unstandardized regression weight = 0.19, standard error = 0.03, *t*_(40.73)_ = 6.17, *p* < 0.001] and the California Verbal Learning Test [unstandardized regression weight = 0.24, standard error = 0.036, *t*_(36.85)_ = 6.71, *p* < 0.001] significantly improved over time. Education significantly predicted Digit Symbol Coding total score above and beyond the other variables [unstandardized regression weight = 0.09, standard error = 0.03, *t*_(71.17)_ = 2.91, *p* = 0.005].

**Table 3:** The relationship of annualized whole brain atrophy with neuropsychological test performance of Extended-CATCH patients.

**Cognitive functioning composite/test score**	**Unstandardized regression coefficient**	**95% CI**	**Standardized regression coefficient**	***p***
Executive functioning composite (*n* = 78 with 195 observations)	0.103	[−0.096, 0.306]	0.076	0.328
Processing speed composite (*n* = 78 with 198 observations)	0.192	[0.006, 0.378]	0.139	0.045
Memory composite (*n* = 78 with 199 observations)	0.008	[−0.128, 0.148]	0.007	0.906
CVLT (*n* = 78 with 205 observations)	−0.013	[−0.155, 0.144]	−0.011	0.87
Boston short form (Total Score) (*n* = 78 with 201 observations)	−0.168	[−0.395, 0.053]	−0.105	0.14
Trail making test—Part A (seconds) (*n* = 78 with 201 observations)	0.081	[−0.159, 0.32]	0.049	0.54
COWA (Total new words) (*n* = 78 with 200 observations)	−0.067	[−0.274, 0.136]	−0.048	0.534
Trail making test—Part B (s) (*n* = 78 with 200 observations)	0.211	[−0.063, 0.488]	0.108	0.169
Digit-symbol coding (Total Score) (*n* = 78 with 200 observations)	0.199	[0.035, 0.366]	0.159	0.019

*COWA, Controlled Oral Word Association Test (total score); CVLT, California Verbal Learning Test (Total recall = total recall over 5 learning trials; Delayed Recall = free recall after 20–25 min); Each row denotes a separate mixed-effects regression model where the indicated cognitive outcome is estimated by atrophy rate and covariates. Only the regression weights, confidence intervals, and p-values for atrophy rate are reported, while the covariates are not. Covariates are: Time of cognitive testing (years), Age (years), Lesion Volume (mL), Education (years), White Matter Hyperintensity Volume (mL), Baseline Gray Matter Volume, Baseline White matter volume. Each Regression model is fit with a random slope and intercept over time for each participant; Bonferroni-adjusted significance level set to p < 0.006*.

*The unstandardized regression coefficients (estimate) are provided for each covariate*.

## Discussion

This study shows that TIA and minor stroke patients experienced higher whole-brain atrophy rates than healthy controls over a 3 years period. In addition, age and presence of diabetes were also significant predictors of whole-brain atrophy rate. But we did not find evidence of a significant effect for history of hypertension, sex, BMI, smoking status, or imaging time interval, or DWI lesion volume, or normalized brain volume on brain atrophy rates. The cognitive profile revealed that subjects who had more delayed processing speed composite scores (WAIS-III Digit-Symbol Substitution) had higher rates of whole brain atrophy. However, these results were not significant after correcting for multiple comparisons. Memory composite and CVLT scores improved over the course of the study, and we found that digital symbol coding was strongly related to educational attainment. These results concur with the previous study of TIA patients that revealed high rates of cerebral atrophy over a year which was associated with higher diastolic blood pressure and white matter hyperintensities (9). Our findings provide further evidence that structural changes in the brain, in this case change in whole brain volume, may underlie a heightened risk of dementia in a TIA/ minor stroke patient population (37) and occurs before deterioration in cognitive tests can be measured. The measurement of whole brain atrophy rate may help identify high-risk patients for targeted intervention. This data expands on previous findings by demonstrating that patients show elevated whole-brain atrophy rates early in the disease progression, before symptoms or signs of cognitive decline develop (38, 39).

The use of rate of brain atrophy may have some advantages as a surrogate marker of preclinical disease progression (40, 41). Rates of brain atrophy have been shown to correlate with cognitive decline and vascular disease and AD (42). Measurement of brain atrophy rate is also more sensitive at predicting cognitive decline and can be measured more precisely than neuropsychological outcomes (43), which are subject to several potential confounders (e.g., baseline cognitive performance, problems with standardization procedures, co-morbid disease and treatment factors, and learning effects). The link between TIA and minor stroke and late life cognitive decline is not known, but a recent study has established that cardiovascular risk factors appear to influence neurodegeneration independent of amyloid markers of AD (44).

The progression of cognitive decline has been reported in patients with stroke. In a recent prospective cohort study, longitudinal telephone cognitive testing in participants aged 45 years or older was conducted pre and post stroke over 6 years (45). Those that experienced stroke were older men with more vascular risk factors, low socio-economic indicators and worse health status, and lower pre-stroke cognition. While acute stroke was associated with acute decline in global cognition in new learning and verbal memory, intriguingly participants with incident stroke later had statistically significant faster declines in global cognition and executive function than in learning or verbal memory when compared to pre-stroke. Another study of stroke and TIA patients detected progressive decline in verbal memory 3 years post stroke in patients without clinical or radiological evidence of recurrent stroke (10). The same study also showed that decline in composite neuropsychological scores was associated with smaller hippocampi, and brain atrophy at 3 years (as a cross-sectional measure) (10). Important potential modifiers of decline in verbal memory and brain atrophy, such as stroke size or location, incipient cerebrovascular disease, presence of *in vivo* AD pathology were not established (46–49). Another recent study selected patients with “TIA presentation” and CT perfusion deficits, and remarkably demonstrated cross-sectional changes in whole brain volume from baseline to 90 days later (11). The paper did not report DWI volume despite reporting moderately large CT perfusion deficits, and changes in cortical gray matter volumes and decline in the Montreal Cognitive Assessment was reported after a short follow-p period of 90 days. Our data adds to these studies by demonstrating improvement in memory and executive composite test scores and relatively modest changes in other cognitive tests using a comprehensive battery despite showing absolute changes in whole brain volume over time.

Expanding on these studies, The Predementia Neuroimaging of Transient Ischemic Attack (PREVENT) prospective longitudinal cohort study is targeting patients presenting with clinically defined motor or language TIA (50). These subjects are generally younger, not demented and in better physical condition than stroke patients, thereby permitting the acquisition of detailed serial neuropsychological assessments in participants that are not disabled and cooperative with cognitive testing, and avoiding high attrition rates caused by stroke related co-morbidity and frailty that can prohibit the study of cognition over time (10). Prospective longitudinal studies design in patients with cerebrovascular presentations utilizing serial high resolution structural MRI and cognitive testing will permit more precise estimates of the relationship of neurodegenerative disease and progressive white matter lesions with rates of cerebral atrophy and cognitive decline than cross-sectional studies. Identifying the disease that contributes to the change in brain volume will be important because the response to preventative treatments may depend on the stage of disease and its pathology. Currently available biomarkers include cerebrospinal fluid AD biomarkers of tau and Aβ_1−42_ that precede cognitive decline and correlate with atrophy (51, 52). Whole brain atrophy is a global, non-specific indicator/marker of diffuse neurodegenerative processes involved in disease such as AD but does not highlight the regional changes that are known to exist through different stages of disease progression in AD patients and cerebrovascular disease (42).

The use of ADNI controls is a potential source of error, however there is precedence for their use in similar comparison studies (53, 54). We were careful to perform regular quantitative and qualitative assessments to optimize signal to noise and reduce image distortion related to uniform and geometric fidelity according to manufacturer specifications. It has been previously demonstrated that scanner variability related to field strength (53, 54) and vendor (55) appears to have minimal effect on such volumetric measurements. There was significant disparity of age and gender between Extended CATCH and HC groups. The ADNI subjects were on average 5 years older than the TIA/ minor stroke group, yet despite being slightly older, they had lower rates of cerebral atrophy across the two-interval time points than the TIA and minor stroke group. Additionally, the measurements of percentage annualized change in brain volume that we have measured in our control subjects conforms with published data (8, 26). The higher proportion of men in the CATCH cohort is expected because men have a higher incidence of TIA and stroke. Nevertheless, gender in our analysis was not a predictor of increased rates of whole brain atrophy. Another potential limitation is the under-diagnosis of hypertension because we restricted our analysis to prior history of hypertension or concurrent antihypertensive treatment. Finally, our study population was compromised by the absence of whole brain atrophy rate measurements in a substantial portion at interval one because of technical reasons related to a MRI system upgrade.

Our findings indicate that patients presenting with TIA and minor stroke demonstrate greater brain atrophy rates than healthy controls over a 3 years post-event period. The elevated atrophy rate in this population was significant beyond the effects of demographic and vascular risk factors. Whole brain atrophy rates in TIA and minor stroke patients appear to be weakly related to cognitive function. However, if neurodegenerative changes continue and precede cognitive symptoms, atrophy rates could be used to assess the efficacy of vascular risk factor-reduction treatments. This would provide a therapeutic window for slowing changes in brain volume that precede cognitive decline in patients at a high risk of dementia.

## Author Contributions

MM: contributed to post processing of imaging data, data interpretation, assisted in writing the manuscript; JU: contributed to the statistical analysis and interpretation of the data, writing the manuscript; MR: contributed to post processing of imaging data and analysis; GZ: contributed to the imaging analysis and interpretation of data, and writing and editing of the manuscript; LG: contributed to the imaging analysis and interpretation of data, and proof reading of the manuscript; RG: performed image analysis, quality control check and edited and proof read the manuscript; AS: contributed to the measurement of white matter lesion segmentation, data collection and proof reading of the manuscript; AA: contributed to the measurement of white matter lesion segmentation, data collection and proof reading of the manuscript; ST: assisted with the data collection, statistical analysis and proof reading of the manuscript; RF: assisted in developing the MRI protocol, data collection and writing of the manuscript; TS and MW: conceived the statistical analysis of the data, and proof read the manuscript. SC and ES: conceived the CATCH study, recruited patients, involved in data collection and assisted in writing the manuscript; CD: contributed to post processing of imaging data, organization of the cognitive data, data interpretation, and edited the final version of the manuscript; PB: conceived the hypothesis and formulated the ideas, recruited patients and carried out clinical workup, collected data, and wrote the manuscript. All authors contributed to the final version of the manuscript.

### Conflict of Interest Statement

The authors declare that the research was conducted in the absence of any commercial or financial relationships that could be construed as a potential conflict of interest.
