# Supplementary material for: Longitudinal Brain Atrophy Rates in Transient Ischemic Attack and Minor Ischemic Stroke Patients and Cognitive Profiles
Source: Front Neurol. 2019 Feb 19;10:18. doi: 10.3389/fneur.2019.00018 (PMC6389669; doi:10.3389/fneur.2019.00018)
Supplement: Supplementary file 1 [file Data_Sheet_1.docx]

Table S1. Median (IQR) z-scores for neuropsychological tests at the four time points. *Significantly different from the baseline time point (ANOVA, *p < 0.05*)

|  | **Baseline** | **1 year** | **2 years** | **3 years** |
| --- | --- | --- | --- | --- |
| Executive Composite | 0.14(-0.53, 0.58) | 0.28(-0.13, 0.83) | 0.29(-0.18, 0.81) | 0.34(-0.28, 0.81) |
| Processing Composite | 0.13(-0.65, 0.72) | 0.38(-0.65, 0.72) | 0.39(-0.22, 0.85) | 0.47(-0.30, 0.92) |
| Memory Composite | -0.04(-0.54, 0.35) | 0.31(-0.26, 0.80) | 0.51(-0.02, 0.87)* | 0.65(0.20, 1.14)* |
| CVLT | -0.21(-0.82, 0.30) | 0.25(-0.37, 0.65)* | 0.35(-0.03, 0.79)* | 0.37 (0, 1.07)* |
| Boston Naming Score | 0.82(0, 0.83) | 0.82(0, 0.83) | 0.39(0, 0.83) | 0.52(0, 0.83) |
| Trail Making Test A | 0.15(-0.65, 0.64) | 0.17(-0.33, 0.73) | 0.08(-0.47, 0.96) | 0.25(-0.52, 0.76) |
| Trail Making Test B | 0.43(-0.54, 0.92) | 0.69(-0.14, 1.13) | 0.56(-0.29, 1.36) | 0.47(-0.44, 1.17) |
| Digit Symbol Coding | 0(-1.0, 0.33) | 0.17(-0.66, 0.66) | 0(-.033, 0.66) | 0.33(-0.33, 0.66)* |
| Controlled Oral Word Association Test A | -0.24(-0.86, 0.41) | 0(-0.73, 0.73) | 0.03(-0.57, 0.59) | 0.19(-0.66, 0.57) |
